# Simultaneous Estimation of Nebivolol Hydrochloride and Valsartan using RP HPLC

**DOI:** 10.4103/0250-474X.54270

**Published:** 2009

**Authors:** S. U. Kokil, M. S. Bhatia

**Affiliations:** Department of Pharmaceutical Chemistry, Bharati Vidyapeeth College of Pharmacy, Kolhapur-416013, India

**Keywords:** Nebivolol hydrochloride, valsartan, fenofibrate, ion-pair, RP-HPLC

## Abstract

In this study, a rapid, precise, accurate, specific and sensitive ion-paired reverse phase liquid chromatographic method has been developed for the simultaneous estimation of nebivolol hydrochloride and valsartan in their capsule formulation. The chromatographic method was standardized using a HIQ sil C_18_ column (250×4.6 mm i.d., 5 μm particle size) with UV detection at 289 nm and flow rate of 1 ml/min. The mobile phase consisting of methanol:water (80:20 v/v) with addition of 0.1 percent 1-hexanesulfonic acid monohydrate sodium salt as an ion-pairing reagent was selected. The method was validated and produced accurate and precise results for estimation of the two drugs.

Nebivolol hydrochloride (NEB, fig. 1), 1-(6-fluorochroman-2-yl)-{[2-(6-fluorochroman-2-yl)-2-hydroxyethyl]amino}ethanol, is a selective β_1_ blocker[1–7]. Literature assessment showed that high performance liquid chromatography (HPLC), high performance thin layer chromatography (HPTLC)[8], and liquid chromatography-mass spectroscopy (LC-MS)[9–11] methods are reported for estimation of NEB in dosage formulations and in biological fluids. Valsartan (VAL, fig. 1), 3-methyl-2-[pentanoyl-[[4-[2-(2H-tetrazol-5-yl)phenyl]phenyl]methyl]amino]butanoic acid, is an angiotensin II receptor antagonist[12]. Both these drugs are used for the treatment of high blood pressure and other cardiovascular pathophysiologic conditions. A LC-MS[13] and HPLC method[14] are reported for estimation of VAL in human plasma. The present research work describes rapid, accurate, sensitive and reproducible RP-HPLC method for simultaneous estimation of NEB and VAL from the capsule formulation.

**Fig. 1:**
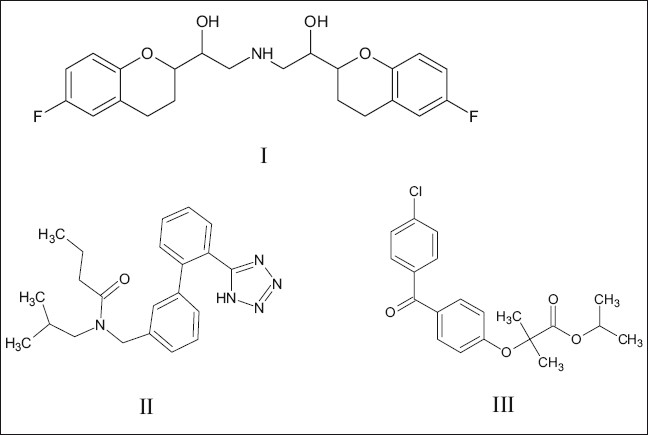
Chemical structures. Chemical structures of I, nebivolol hydrochloride, II, valsartan and III, fenofibrate

The HPLC system used was a computer based Jasco series instrument comprising of a pump PU-2080 and a UV detector UV-2070. Manual injections were carried out using a Rheodine injector with a fixed 20 μl external loop. The chromatographic separations were performed on a HIQ sil C_18_ ODS column (250×4.6 mm i.d., 5 μm particle size), operating at ambient temperature, using a mobile phase consisting of methanol:water (80:20 v/v) with addition of 0.1 percent 1-hexanesulfonic acid monohydrate sodium salt as an ion-pairing reagent. The mobile phase was filtered through 45μm nylon membrane filter. A Shimadzu AY 120 analytical balance was used for weighing. A PCi ultrasonicator was used for sonication. Mobile phase was used for preparation of drug samples throughout the analysis.

HPLC grade methanol and 1-hexanesulfonic acid monohydrate sodium salt were purchased from Loba Chem. Pure drug samples of NEB and VAL was kindly supplied as gift samples by Emcure Pharm. Ltd., Pune, India and Lupin Ltd., Mumbai, India, respectively. Fenofibrate (FEN, fig. 1) was kindly supplied as gift sample by Cipla Ltd., Mumbai, India. Double distilled water filtered through 45 μm cellulose nitrate membrane filter used for analysis.

Standard stock solutions containing NEB and VAL were prepared by transferring 25 mg of NEB and VAL separately into two 25 ml volumetric flasks. It was then dissolved in 15 ml of mobile phase and ultrasonicated for 10 min. Final volume of the solution was made up to 25 ml with mobile phase to get stock solutions containing 1000 μg/ml of NEB and VAL. FEN, was selected as an internal standard. Standard stock solution containing FEN was prepared by dissolving 25 mg of FEN in 15 ml of mobile phase in a 25 ml volumetric flask. It was ultrasonicated for 10 min and then final volume of solution was made up to 25 ml with mobile phase to get final concentration as 1000 μg/ml of FEN.

The solutions having concentrations of 10-250 μg/ml of NEB and 160-400 μg/ml of VAL were prepared by proper dilutions of primary stock solutions of pure drugs with mobile phase to obtain working standards. To each 10 ml volumetric flask 0.4 ml of FEN was added as an internal standard and the final volume was made up with the mobile phase. A 20 μl of sample solution was injected into the chromatographic system using fixed volume loop injector and chromatograms were run for 15 min acquisition. The flow rate was maintained at 1 ml/min at ambient temperature and the eluents were monitored at 289 nm. The separation was done on a C_18_ column using developed mobile phase which contains methanol:water (80:20 v/v) with 0.1 percent 1-hexanesulfonic acid monohydrate sodium salt.

Marketed capsule formulation containing a NEB 5 mg and VAL 80 mg was procured from a local pharmacy and was analyzed using this method. Twenty capsules of formulation were accurately weighed and average weight calculated. The capsule shell was then opened to collect two tablets and triturated. An accurately weighed triturated powder equivalent to 5 mg of NEB and 80 mg VAL of the formulation was transferred to a separate 50 ml volumetric flask containing 25 ml of mobile phase. Prepared solution was sonicated for 10 min for proper solubilization of the drug and final volume was made up to 50 ml with mobile phase. This solution was then filtered through Whatman filter paper No. 41. Three aliquots in suitable concentration were prepared in triplicate to get nine solutions. Each solution was filtered through hydrophilic PVDF 45 μm size syringe filter. Filtered aliquots were analysed using proposed method.

Accuracy of analysis was determined by performing recovery studies by spiking different concentrations of NEB and VAL in the pre analyzed formulation samples within the analytical concentration range of the proposed method.

An ion-pair RP-HPLC method was developed for estimation of NEB and VAL, which can be conveniently employed for routine quality control in pharmaceutical dosage forms. The chromatographic conditions were optimized in order to provide a good performance of the assay. In order to affect the simultaneous elution of the two components under isocratic conditions, different chromatographic conditions (organic modifier, flow rate, ionic strength, and pH) were investigated. RP-HPLC system consisting of HIQ sil C_18_ column (250×4.6 mm i.d.) provided good resolution for separation of NEB and VAL. Initially the mobile phase for the two drugs was selected based on its polarity. Mobile phase containing methanol alone or acetonitrile alone was found to elute the two compounds unresolved. Various ratios of methanol:acetonitrile:water were found to produce chromatograms with very close retention times. The cationic nature of NEB produced broad asymmetrical peaks with aqueous-organic mobile phases due to the ionic interaction of the charged solutes with the free silanol groups on the alkyl bonded reversed phase packings. Use of ion-pairing agents like 1-hexanesulfonic acid monohydrate sodium salt and quaternary amine like triethylamine is found to improve separation chromatographic peaks and decrease their asymmetries. Taking the above phenomenon into account for this analysis 0.1 percent 1-hexanesulfonic acid monohydrate sodium salt was selected and better resolution was achieved when the concentration of ion-pairing regent was maintained between 0.1-0.2 percent. Best resolution was achieved at the mobile phase composition of methanol:water in the ratio of 80:20 v/v with 0.1% 1-hexanesulfonic acid monohydrate sodium salt, where the peaks of NEB, VAL and FEN were distinctly resolved. Flow rate of 0.5 ml/min resulted in greater retention times and 1.2 ml/min resulted in very close retention times with poor resolution. A flow rate of 1 ml/min resulted in elution of all drugs within 15 min with good resolution.

The sampling wavelength was selected after scanning the drug solutions in the mobile phase having concentration of 10 μg/ml in the UV range of 200-400 nm on a UV spectrophotometer. 289 nm was selected as suitable wavelength for estimation. FEN was found to be a suitable internal standard for this study under the selected chromatographic conditions. The calibration curves exhibited good linearity. The response factors for concentrations 10, 50, 90, 130, 170, 210, 250 μg/ml of NEB were found to be 0.0668, 0.2946, 0.5300, 0.8021, 1.0683, 1.3057, and 1.5495 and for concentrations 160, 200, 240, 280, 320, 360, 400 µg/ml of VAL were found to be 0.2301, 0.3167, 0.3969, 0.4971, 0.5479, 0.6155, and 0.6903, respectively. The overlain chromatograms are shown in fig. 2. Corresponding regression equations were generated from which slope, intercept and coefficient of correlation values for calibration curve were obtained. The correlation coefficients, slopes and intercepts were found to be 0.9998, 0.0062 and -0.0043 for calibration curve of NEB and 0.9979, 0.0017 and -0.0092 for calibration curve of VAL, respectively. The statistical data obtained for the estimation of NEB and VAL in capsule formulations by the proposed method is shown in Table 1. Results of recovery studies indicated that the method is rapid, accurate, sensitive and reproducible. The statistical data for the recovery studies is given in Table 2.

**Fig. 2:**
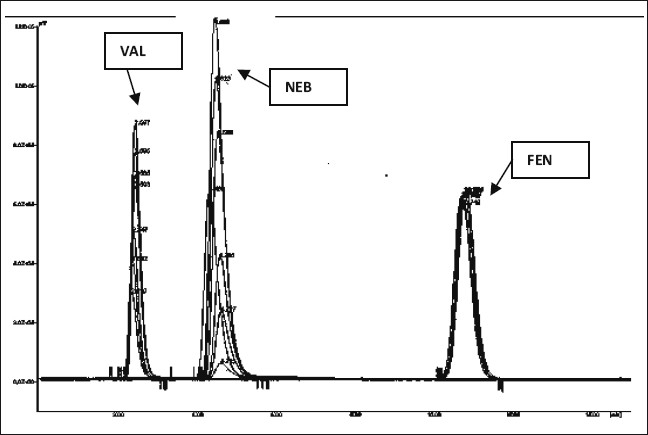
Overlain of chromatogram of NEB, VAL and FEN. Overlain chromatograms of nebivolol hydrochloride NEB, valsartan VAL and fenofibrate FEN

**TABLE 1:** ANALYSIS OF CAPSULE FORMULATION

Formulation	Analyte	Label claim (mg)	Percent label claim estimated* (Mean±SD)
Capsule	NEB	5	99.82±0.34
	VAL	80	99.87±0.32

NEB denotes nebivolol hydrochloride while VAL denotes valsartan.

* Indicate average of nine determinations and SD denotes standard deviation.

**TABLE 2:** RECOVERY STUDIES

Formulation	Analyte	Label claim (mg)	Percent label claim estimated* (Mean±SD)
Capsule	NEB	5	99.05±1.01
	VAL	80	99.08 ±1.05

NEB denotes nebivolol hydrochloride while VAL denotes valsartan.

* Indicate average of nine determinations and SD denotes standard deviation.

The system suitability parameters such as theoretical plates, asymmetry, capacity factor, tailing factor and resolution were found to be 1544.14 and 1906.56, 1.48 and 1.65, 465.00 and 257.50, 1.27 and 1.24, 4.91 and 5.96 for NEB and VAL, respectively. The method was specific as none of the excipients interfered with the analytes of interest. The proposed method was applied to the determination of NEB and VAL in their pharmaceutical preparation. The results indicate satisfactory accuracy and precision of the method.

The method was validated according to ICH guidelines[15]. The limit of detection has been calculated as DL=3.3σ/S, where, DL is detection limit, σ is the residual standard deviation of the response and S is the slope of the calibration curve. The limit of detection with calibration curve was found to be 2.28 μg/ml for NEB and 17.58 μg/ml for VAL.

The limit of quantitation has been calculated as QL=10σ/S, where, QL is quantitation limit, σ is the standard deviation of the response; S is the slope of the calibration curve. The limit of quantitation, with calibration curve was found to be 6.92 μg/ml for NEB and 53.29 μg/ml for VAL. A calibration curve was plotted using response factor obtained against respective concentration of NEB and VAL. The linear regression data for the calibration curves showed good linear relationship over the concentration range of 10-250 μg/ml for NEB and 160-400 μg/ml VAL with coefficient of correlation.

The accuracy of proposed method was determined by investigating the recovery of NEB and VAL at three levels, ranging from 80 to 120% of the theoretical concentration, from placebo mixtures spiked with active substances. It showed mean percent recovery of 99.05±1.01 for NEB and 99.08±1.05 for VAL.

The precision and accuracy of the developed method were expressed in terms of relative standard deviation (RSD). The result depicted reveals excellent accuracy and high precision of the assay method. The intra-day and inter-day variations were evaluated by comparing the slopes of the calibration curve over the concentration range of 10-250 μg/ml for NEB and 160-400 μg/ml VAL. There was no significant variation in the slope values. The mean of RSD was found to be 0.0464 and 0.3958, respectively for intra-day analysis and inter-day analysis of NEB. The same parameter was found to be 0.0759 and 0.5595, respectively for intra-day analysis and inter-day analysis of VAL. Reproducibility of the method was ascertained by varying the analysts and the analysis of variance (ANOVA) yielded correlation coefficient, squared correlation coefficient, standard deviation of residual from line, P-value, slope, Y-intercept and X-intercept values of 0.9997, 0.9994, 0.0134, 0.0001, 0.0062, -0.0100 and 1.607 for NEB and 0.9990, 0.9993, 0.0128, 0.0046, 0.0019, -0.0617 and 3.2456 for VAL, respectively.

The developed method can be suitable for the determination of NEB and VAL in concentration range 10-250 μg/ml and 160-400 μg/ml, respectively. The specificity was determined by comparing test results from the analysis solutions containing active substances. The method allows active compounds to be separated and thus established that it is suitable or specific for desired separation. The stability of solution has been demonstrated by analysing solution of NEB and VAL after 16 h if stored at room temperature; the method is thus suitable for automation. From above data we have concluded that the proposed method is accurate and precise and can be applicable for the in process quality control for determination of NEB and VAL in pharmaceutical preparations.
